# Structure–Activity Relationship of an All-α-helical Prenyltransferase Reveals the Mechanism of Indole Prenylation

**DOI:** 10.1021/acs.biochem.5c00329

**Published:** 2025-09-19

**Authors:** Takumi Oshiro, Shuta Uehara, Arisa Suto, Yoshikazu Tanaka, Takuya Ito, Yoshio Kodera, Takashi Matsui

**Affiliations:** † Department of Physics, School of Science, 12877 Kitasato University, Sagamihara, Kanagawa 252-0373, Japan; ‡ Graduate School of Life Science, 13101 Tohoku University, Sendai, Miyagi 980-8577, Japan; § Faculty of Pharmacy, 38307 Osaka Ohtani University, Tondabayashi, Osaka 585-8540, Japan; ∥ Center for Disease Proteomics, School of Science, Kitasato University, Sagamihara, Kanagawa 252-0373, Japan

## Abstract

Enzymes are involved in the biosynthesis of a variety of secondary metabolites found in nature. The catalytic mechanism is regulated by the three-dimensional structure of the enzyme, particularly at the catalytic site, resulting in the synthesis of natural products with complex conformations derived from a regioselective, chemoselective, or stereoselective preference of the enzyme reaction. Prenyltransferase, which belongs to the prenylsynthase superfamily, catalyzes the condensation of isoprene to an aromatic compound, consequently producing a terpenoid scaffold structure. Prenyltransferase thus plays an important role in expanding the chemical diversity of the terpenoids. Although the three-dimensional structures of prenylsynthases categorized in the same superfamily have been resolved, the catalytic mechanism of prenyltransferase has been veiled. In this study, we determined the X-ray crystal structure of a novel prenyltransferase, Ord1, which is derived from *Streptomyces*. Here, we report the enzymatic characteristics of the Ord1 and discuss its catalytic mechanism.

## Graphical abstract

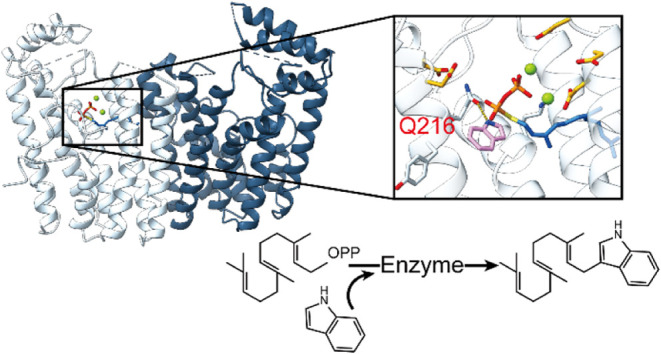

## Introduction

To date, more than 440,000 natural compounds have been identified; these compounds are ubiquitous across all domains of life and exhibit a wide range of biological activities.^1,2^ Terpenoids, which represent the largest and most widely distributed family of natural products,^3^ play a variety of important physiological roles in nature, such as maintaining membrane stability, facilitating photosynthesis, and providing chemical defense.^4^ Terpenoids are widely used in products such as perfumes, pharmaceuticals, and fuels.^3,5,6^ The enormous variety of structures derived from diverse terpene scaffolds is involved in the enzymes of the prenylsynthase superfamily, which includes synthases, cyclases, and transferases. In primary metabolism, a prenylsynthase condenses an isopentenyl pyrophosphate (IPP, C_5_) with a dimethylallyl pyrophosphate (DMAPP, C_5_) to generate a geranyl pyrophosphate (GPP, C_10_). Some prenylsynthases can accommodate isoprenoids with longer chains and thus condense additional rounds of IPP to elongate isoprene units and generate longer isoprenoids, such as farnesyl pyrophosphate (FPP, C_15_) and geranylgeranyl pyrophosphate (GGPP, C_20_).^7–9^ Some isoprenoids synthesized by prenylsynthase are cyclized by prenylcyclase to form compounds used in perfumes, such as γ-terpinene.^10^ The three-dimensional structures and catalytic mechanisms of prenylsynthase and prenylcyclase are well-known.^11–13^ These enzymes consist entirely of α-helices and share similar catalytic mechanisms.^8,11^ Additionally, they contain a highly conserved metal ion coordinating domain (DDXXD), referred to as the aspartate-rich motif (ARM).^8,12^

Other isoprenoids often serve as starter substrates to produce various indole alkaloids. For instance, PaxC, an ABBA-type indole prenyltransferase, condenses GGPP to an indole-3-glycerol phosphate to yield a key intermediate in the biosynthesis of paxillin.^14^ In addition, ABBA-type enzymes exhibit promiscuous substrate recognition and thereby generate a wide variety of products; these enzymes share a similar antiparallel αββα barrel conformation.^15^ Structure-based engineering of ABBA-type enzymes resulted in increased substrate acceptance^16^ and facilitated the production of a diverse range of novel compounds by providing different substrates.^17^ UbiA-type enzymes, a membrane-embedded prenyltransferase, accommodate a broad range of substrates, ranging from DMAPP (C_5_)^18,19^ to dodecaprenyl phosphate (C_60_)^20^ as a prenyl donor substrate, and this enzyme is involved in the biosynthesis of ubiquinone.^18,19,21^ UbiA-type enzymes have two characteristics conserved motifs, NDXXDXXXD and DXXXD,^22^ similar to those found in all-α-helical prenylsynthase. Analysis of the three-dimensional structure of UbiA revealed how the prenyltransferase reaction proceeds.^18,19^ Although the synthase forms a soluble homodimer, UbiA is a monomeric membrane protein.

According to the proposed biosynthetic mechanism of indolosesquiterpenoid, another type of prenyltransferase, XiaM, mediates the biosynthesis of xiamycin A.^23–25^ This enzyme shows higher sequence similarity to prenylsynthases and cyclases than other types of transferases (e.g., ABBA-type transferase) and shares an ARM, suggesting it is a member of the all-α-helical prenylsynthase superfamily, all α-helical prenyltransferase. A recent in silico study supplemented by the use of an AlphaFold2-informed protein model^26^ predicted that all α-helical prenyltransferase prenylates polypeptides through a dissociative electrophilic substitution mechanism.^27–30^ Previous research has shown that all-α-helical type prenyltransferases possess a first ARM (FARM), whereas the region corresponding to the second ARM (SARM) is less conserved and thus designated a pseudo-SARM.^31^ Furthermore, previous mutational studies have suggested that FARMs and pseudo-SARMs play important roles in enzyme reactions.^26,31,32^ However, the three-dimensional structures of all-α-helical prenyltransferases have remained elusive, and the mechanism of prenylation catalyzed by this type of enzyme is unclear.

In this study, we focused on all-α-helical prenyltransferase Ord1, which is derived from *Streptomyces* sp. KS84. Ord1 may be involved in the biosynthesis of the oridamycins A and B,^33^ highly shared conformation with xiamycin A.^23,24^ Ord1 purportedly functions as a prenyltransferase due to the presence of a conserved FARM and pseudo-SARM, and the enzyme exhibits high sequence homology to XiaM (79% identity), which catalyzes the biosynthesis of xiamycin A.^25^ Here, we report the X-ray crystal structure of Ord1 and discuss the mechanism of prenylation by Ord1.

## Materials and Methods

### Protein Expression and Purification of Ord1 and Its Mutants

The draft sequence of *Streptomyces* sp. KS84 genome was obtained using a Genome Analyzer II (Illumina Inc., San Diego, CA). Chromosomal DNA (5.0 μg) was used for library construction and sequencing according to the procedure recommended by Illumina Inc. DNA assembly was performed using CLC genomic workbench software 5.1 (CLC Bio). The *ord1* gene was cloned into a pET-26b vector (Merck, Darmstadt, Germany), and Ord1 fused with a C-terminal His_6_ tag was overexpressed in *Escherichia coli* strain Rosetta2 (DE3) (Merck) under the control of a T7 promoter. Cells harboring the plasmid were grown in LB medium containing 20 mg/L kanamycin and 25 mg/L chloramphenicol at 37 °C until the optical density at 600 nm (OD_600_) reached 0.6. 0.125 mM isopropyl-β-d-1-thiogalactopyranoside (IPTG) was then added to induce Ord1 expression, and the culture was continued for 15 h at 20 °C. The cells were harvested by centrifugation at 8000*g* for 15 min at 4 °C and resuspended in 40 mL of lysis buffer (50 mM Tris–HCl [pH 8.0], 300 mM NaCl, 10% [v/v] glycerol). The resulting cell lysate was centrifuged at 8000*g* for 15 min at 4 °C, and the supernatant was passed through a 5-μm filter device (Merck) and loaded onto a 1 mL HisTrap HP column (Cytiva, Marlborough, MA) equilibrated with lysis buffer. The recombinant protein was eluted with lysis buffer containing 300 mM imidazole. The protein solution was further purified to homogeneity using size-exclusion chromatography on a HiPrep 16/60 Sephacryl S-200 column (Cytiva) equilibrated with lysis buffer. Appropriate fractions were analyzed for purity using sodium dodecyl sulfate-polyacrylamide gel electrophoresis (SDS-PAGE). The fraction containing Ord1 was concentrated to 12 mg/mL (based on measurement of the UV absorbance at 280 nm) using an Amicon Ultra 15 centrifugation device (Cytiva).

Selenomethionine-labeled Ord1 (SeMet-labeled Ord1) was overexpressed in *E. coli* strain Rosseta2 (DE3). The cells were grown at 37 °C in M9 medium containing 1 g/L NH_4_Cl, 1 mM MgSO_4_, 0.1 mM CaCl_2_, 1× Basal Medium Eagle (BME) vitamins solution (Merck), 0.4% (w/v) glucose, 20 mg/L kanamycin, and 25 mg/L chloramphenicol until the OD_600_ reached 0.6. Next, 25 mg/L SeMet and 100 mg/L each of l-Lys, l-Thr, l-Ile, l-Leu, l-Val, and l-Phe were added, and the cells were cultured for an additional 30 min at 37 °C. Subsequently, 0.125 mM IPTG was added to induce the expression of SeMet-labeled Ord1, and the culture was continued for 15 h at 20 °C. Purification of SeMet-labeled Ord1 was performed as described above.

### Docking Simulation

The QuickPrep in MOE (version 2020.09, Chemical Computing Group, Montreal, Quebec, Canada) was used for refinement, protonation, and energetic minimization of apo-Ord1 WT. The indole molecule was docked into the QuickPrep-treated Ord1 structure using the MOE template-docking protocol. The binding pocket was predicted by following the complex structure of prenylsynthases bound with substrates and the Ord1–FSPP complex. Default parameters of the docking protocol in Schrodinger’s suite were used, and the induced fit refinement approach was applied to generate docking positions during the docking calculation.

### Site-Directed Mutagenesis

Plasmids encoding Ord1 mutant enzymes were constructed using a KOD-Plus-mutagenesis kit (TOYOBO, Osaka, Japan), according to the manufacturer’s protocol using the Ord1 expression plasmid as a template. Tyr215 was substituted with Phe (to give Ord1 Y215F); Tyr215 was substituted with Ala (to give Ord1 Y215A); Gln216 was substituted with Ala (to give Ord1 Q216A); and Asp219 was substituted with Ala (to give Ord1 D219A). The mutant enzymes were expressed and purified according to the same procedures described above for wild-type (WT) Ord1. All primers used to generate Ord1 mutants are listed in Table S1.

### Ord1 Enzyme Assay

To assess prenylindole-forming activity, Ord1 or mutant enzyme (30 μM) was coincubated at 20 °C for 12 h with 1 mM FPP, 1 mM imidazole, and 5 mM MgSO_4_ in 100 μL of 100 mM Tris–HCl (pH 8.0). The enzyme reaction products were extracted using an equal volume of ethyl acetate. The organic layer was analyzed using high-performance liquid chromotagraphy (HPLC) (Nanospace SI-2, Shiseido, Tokyo, Japan), and the enzyme reaction products were eluted on a CAPCELL PAK C18 BB-H column (2.1 mm ID × 100 mm; OSAKA SODA, Osaka, Japan) at a flow rate of 0.2 mL/min using a gradient of mobile phase A (0.01% formic acid) and mobile phase B (90% CH_3_CN, 0.001% formic acid) as follows: 0–6 min, 10% B; 6–28 min, 10–100% B; 28–50 min, 100% B.

The products were analyzed by liquid chromatography-mass spectrometry (LC-MS) using a Q-Exactive quadrupole Orbitrap benchtop mass spectrometer (Thermo Fisher Scientific) equipped with a Nanospace SI-2 HPLC (Shiseido). The enzyme reaction products were injected directly onto a CAPCEL PAK C_18_ BB-H column operated at a flow rate of 200 μL/min and separated using a gradient of mobile phases A and B as follows: 0–2 min, 30% B; 2–8 min, 30–100% B; 8–18 min, 100% B. The analytical conditions were as described previously^34^ with slight modifications. MS1 spectra were collected over the scan range 200–700 *m*/*z* at 140,000 resolution to hit an automatic gain control target of 1 × 10^6^.

To assess the cyclase activity, 30 μM Ord1 was incubated with 1 mM FPP and 5 mM MgSO_4_ in 100 μL of 100 mM Tris–HCl (pH 8.0) at 20 °C for 12 h. The enzyme reaction products were extracted using an equal volume of ethyl acetate. The organic layer was analyzed as described above for the assessment of prenylindole-forming activity.

To assess synthase activity, 30 μM Ord1 was incubated with 2 mM FPP or 2 mM GPP in 100 μL of 100 mM Tris–HCl (pH 8.0), 2 mM IPP and 10 mM MgSO_4_ at 20 °C for 12 h. An equal amount of 30 μM Ord1 with 1 mM indole was then added, and the reaction was continued for a further 12 h at 20 °C. After extraction of the reaction products using an equal volume of ethyl acetate, the organic layer was analyzed by HPLC as described above. The product was directly injected into the atmospheric pressure ionization source operating in heated electrospray ionization mode using a syringe pump at a flow rate of 5 μL/min. The measurement parameters of the Orbitrap Exploris 240 mass spectrometer (Thermo Fisher Scientific) were set as follows: spray voltage, 3.2 kV; transfer tube temperature, 320 °C; scan range, 200–300 *m*/*z* at 60,000 resolution.

### Determination of the Crystal Structures of Ord1 and Mutant Enzymes

All crystallizations were performed by using the sitting-drop vapor diffusion technique. Diffraction-quality crystals of Ord1, SeMet-labeled Ord1, and the Q216A and D219A mutant enzymes appeared after a few days at 18 °C. All crystallization drops were prepared by mixing 0.5 μL of the respective purified protein solution with an equal volume of reservoir solution and equilibrating the mixture against 50 μL of reservoir solution. To obtain a substrate complex structure, Ord1 or mutant enzyme crystal was soaked for 24 h at 20 °C in reservoir solution containing 1 mM farnesyl *S*-thiolodiphosphate (FSPP), 5 mM indole, and 5 mM MgSO_4_.

The diffraction-quality crystals were transferred to a reservoir solution containing 10% (v/v) ethylene glycol and then flash cooled in liquid nitrogen. X-ray diffraction data sets were collected using the BL-1A beamline at the Photon Factory (Tsukuba, Japan) for crystals of Ord1 WT, Ord1-FSPP, and Ord1 Q216A-FSPP, the BL-5A beamline at the Photon Factory for crystals of SeMet-labeled Ord1, and the BL-17A beamline at the Photon Factory for crystals of Ord1 D219A. A wavelength of 0.97922 Å was used for the single-wavelength anomalous diffraction phasing, whereas a wavelength of 1.04500 Å was used for Ord1 WT, Ord1-FSPP, and Ord1 Q216A-FSPP, and 0.98000 Å was used for Ord1 D219A.

Diffraction data for single-wavelength anomalous diffraction analysis^35^ was processed and scaled using the XDS^36^ program package. Selenium (Se) sites were determined using Autosol in PHENIX.^37^ The sites were refined, and the initial phases were calculated using AutoBuild in PHENIX.^38^ The initial phases of the Ord1 apo structure were determined by molecular replacement using the structure of SeMet-labeled Ord1 as a search model. Phenix.xtriage^39^ identified the crystals of Ord1 WT, Ord1-FSPP, Ord1 Q216A-FSPP, and Ord1 D219A as pseudomerohedral twins. Twin law operators^39^ were determined after confirming the correct space group. Molecular replacements were performed using Ord1 WT as the search model with Molrep in CCP4 suite^40^ to solve the complex structure with FSPP and the mutant enzymes. The structures were modified manually using COOT^41^ and refined with Phenix.Refine.^42^ The twin operator (*h*, −*k*, *h–l*) was applied during each refinement for Ord1 WT, Ord1-FSPP, and Ord1 Q216A-FSPP. For Ord1 D219A, the twin operator (*h*, −*k*, −*h–l*) was applied in all refinement cycles. Details regarding data collection and refinement statistics are summarized in Table S2. The quality of the final models was assessed using Molprobity.^43^ All crystallographic figures were prepared using PyMOL^44^ and ChimeraX.^45^ Coordinates and structure factors for Ord1 WT, Ord1-FSPP, Ord1 Q216A-FSPP, and Ord1 D219A were deposited in the Protein Data Bank as entries 9V4G, 9V4J, 9V4H, and 9V4I, respectively.

## Results and Discussion

### Structure of Ord1

To understand the structural basis of substrate recognition, the crystal structure of SeMe-labeled Ord1 was determined at a resolution of 1.8 Å using Se single-wavelength anomalous dispersion. The crystal structure of apo-Ord1 was solved at 2.1 Å resolution using the molecular replacement method with SeMet-labeled Ord1 as the search model (Figure 1). An asymmetric unit of the apo-Ord1 crystal contained two homodimers formed by molecules A–B and molecules C–D (Figure 1A). The overall structure of Ord1 was found to be composed of 16 α-helices. Molecules B, C, and D exhibited root-mean-square deviation (RMSD) values for molecule A of 0.2, 0.9, and 1.0 Å, respectively. Molecule D also converged well with molecule C, with an RMSD value of 0.8 Å, suggesting that the homodimer composed of molecules A and D has a similar overall conformation with the other homodimer composed of molecules B and C.

**Figure 1:**
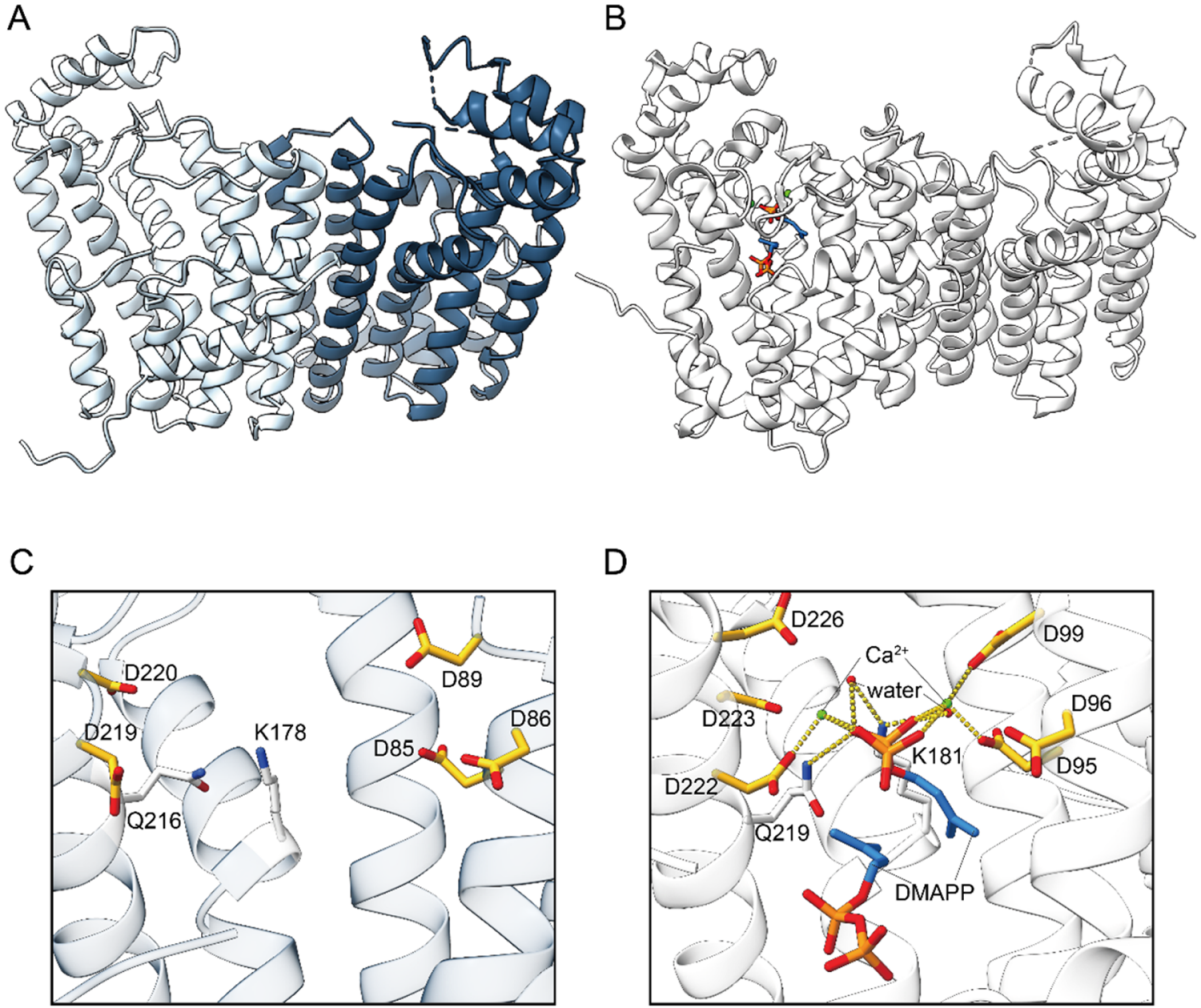
Comparison of crystal structures of apo-Ord1 and the prenylsynthase 3OYR. The overall structures of (A) Ord1 and (B) 3OYR (PDB ID: 3OYR) are illustrated as ribbon diagrams. The structure of Ord1 is colored according to the protomer. The conserved ARMs are compared for (C) Ord1 and (D) 3OYR. Side chains of the aspartic acid residues in the motifs are shown as yellow sticks. Coordination of divalent ions is indicated by yellow dotted lines.

An exploration of structurally similar enzymes using Dali^46^ indicated that several prenylsynthases, such as EcOPPs (PDB ID 3WJO, 26.6% identity with Ord1) and PATL_3739 (PDB ID 4JXY, 27.6% identity with Ord1), showed RMSD values for Ord1 of 2.4 Å (*z*-score 29.2) and 2.1 Å (*z*-score 29.4), respectively (Figure S1). Another prenylsynthase (PDB ID 3OYR, no unique name has been assigned, thus referred to as 3OYR in this paper, Figure 1B) also exhibited an RMSD value for Ord1 of 1.9 Å, with a *z*-score of 30.4. Prenylsynthases commonly contain two ARMs (a FARM and a SARM) that are responsible for condensing the isoprene units, and they may contain a deeper cavity behind the FARM depending on the length of the accommodated chain. The three aspartic acid residues in the FARM of prenylsynthases are essential for pyrophosphate recognition. Three aspartic acid residues in Ord1 (D85, D86, and D89) are conserved with other prenylsynthases, such as 3OYR, and form FARM (Figure 1C,D). Ord1 also contains a deeper cavity behind the FARM, as seen in other prenylsynthases (Figure 2A). To estimate the length of the prenyl chain accommodated by the enzyme, the cavity of Ord1 was compared with those of E-PTS (PDB: 3PDE, 29.2% identity with Ord1), PaFPPS (PDB 3ZOU, 31.9% identity with Ord1), 3OYR, and *Sinapis alba* GGPP synthase (PDB 2J1P, 31.3% identity with Ord1), which accommodate DMAPP, GPP, FPP, and GGPP, respectively. Figure 2B–E shows that the cavity of Ord1 was deeper than that of PaFPPS; thus, Ord1 readily accommodates one molecule of GPP (Figure 2C). Although the superimposition of FPP bound with 3OYR onto Ord1 showed that the terminus of the prenyl chain clashes with the cavity in Ord1, the cavity is elongated in the other direction relative to the superimposed chain and of sufficient volume to accommodate the tip of the chain (Figure 2D). These data suggest that Ord1 can accommodate one molecule of FPP by adapting the chain trace to the shape of the cavity. GGPP seems to fit well into the cavity of Ord1, whereas the opposite terminus of the substrate, pyrophosphate, is far from the pyrophosphate-recognizing residues (D85, D86, and D89), with a distance >10 Å (Figure 2E). These structural comparisons suggest that Ord1 may recognize and bind to FPP as the longest-chain prenyl donor substrate that it can accommodate.

**Figure 2:**
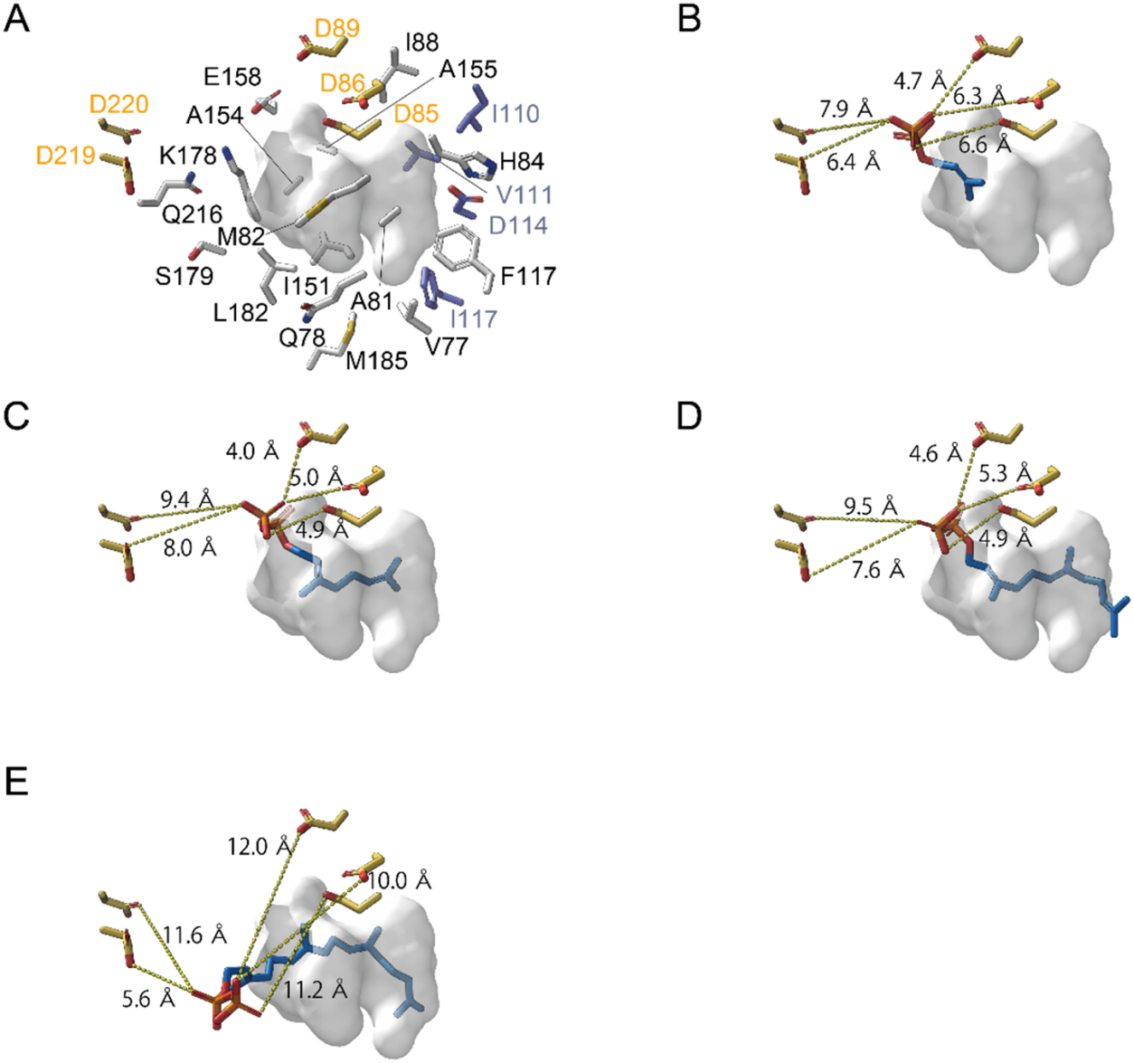
Predicted substrate-binding pocket of Ord1. (A) Maximum chain length of the prenyl moiety accommodated in the substrate-binding pocket of Ord1 predicted by superimposing the structure of apo-Ord1 on previously reported prenylsynthase structures complexed with prenyl pyrophosphates. The predicted substrate-binding pocket of Ord1 is shown as a gray surface model, and the side chains forming this pocket are shown as sticks. (B–E) Superpositions of Ord1 with (B) DMAPP-bound with E-PTS (PDB 3PDE), (C) GPP-bound with PaFPPS (PDB 3ZOU), (D) FPP bound with 3OYR (PDB 3OYR), and (E) GGPP-bound with *S. alba* GGPP synthase (PDB 2J1P). The respective prenyl pyrophosphates from these structures are positioned within the predicted pocket of Ord1. Yellow dashed lines indicate interactions between pyrophosphate groups and the Oδ atoms of aspartic acid residues in the ARMs.

### Complex Structure of Ord1 Bound to the Substrate

Based on comparisons of the structure of Ord1 with those of other prenylsynthases (Figure 2), the crystal of apo-Ord1 was soaked in a solution containing an FPP analog (FSPP). The complex structure of Ord1-FSPP was solved via molecular replacement analysis using apo-Ord1 as a search probe. There were two homodimers in an asymmetric unit of Ord1-FSPP as seemed in the apo-Ord1 (Figure 3A). The RMSD value between the apo- and FSPP complexes was 0.4 Å (Figure 3B). FSPP fit well into the cavity of Ord1, and the C1 atom of FSPP in complex with Ord1 was located 3 Å closer to residue D219 in the pseudo-SARM than in 3OYR, suggesting that FPP is the longest chain of the prenyl donor substrate accepted by Ord1 (Figure 3C).

**Figure 3:**
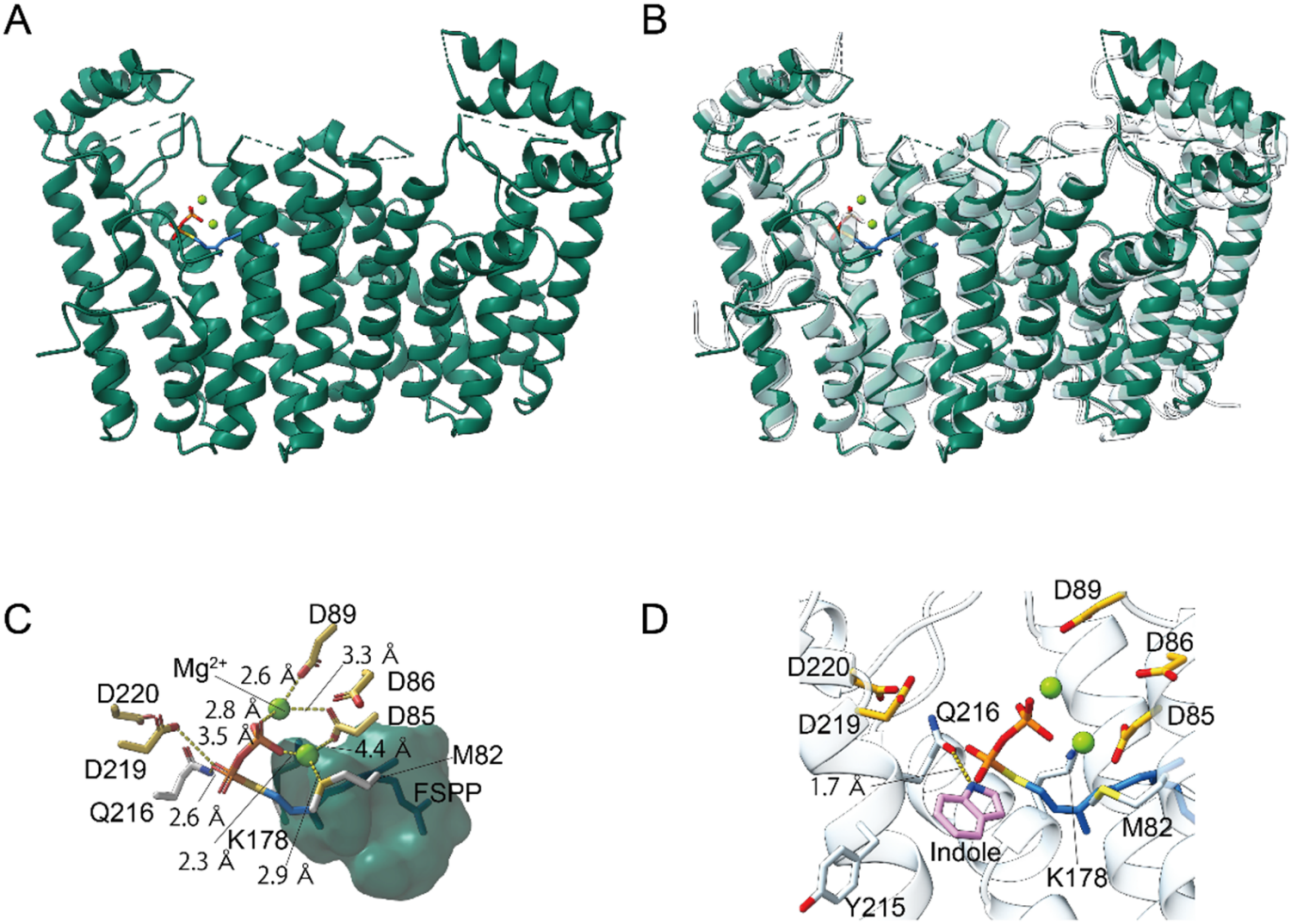
Complex structure of Ord1 bound with FSPP. (A) Overall structure of Ord1-FSPP illustrated as a ribbon diagram. (B) Structural comparison of bound FSPP (green) and apo forms (white) is shown. (C) Close-up view of the substrate-accommodation pocket is shown. The side chains of aspartic acid residues and FSPP and Mg^2+^ ions are indicated as yellow, blue, and green spheres, respectively. The accommodated pocket is indicated as a surface model drawn in green. The D219 residue in the SARM and the D85 and D89 residues in the FARM interact directly with FSPP via hydrogen bonds and indirectly with FSPP via the coordination of Mg^2+^ ions, respectively. (D) A docking model simulated using MOE is shown. The simulated position of the indole molecule is superimposed onto the complex structure of Ord1-FSPP. The areas between the N1 atom of indole and the Oε atom of Q216 are indicated by yellow dotted lines.

Compared to previously determined structures of prenylsynthases, two Mg^2+^ ions could coordinate with D85 and D89 in the FARM and pyrophosphate (Figure 3C). Additionally, the nonconserved residue M82 of Ord1 interacted with pyrophosphate via one Mg^2+^ ion. The α-phosphate of FSPP is located close to the Nε atom of Q216, at a distance of 2.6 Å, enabling the formation of a hydrogen bond; the interactions between the FARM residue D86 and K178 and α-phosphate have been lost. Although another Mg^2+^ ion is coordinated with the SARM residues (D222, D223, and D226) of 3OYR and the pyrophosphate of DMAPP, no metal ions were found around the pseudo-SARM residues (D219, D220, and A223) of Ord1, and a hydrogen bond was identified between the Oδ atom of D219 and α-phosphate (Figure 3C).

Structural comparisons between FSPP-bound and FSPP-free Ord1 revealed that the side chain of Y215, which is located close to the pseudo-SARM, is flipped by 135.5° (Figure S2), thereby creating a space around the pyrophosphate that is sufficient to accommodate a prenyl acceptor substrate. The sequence similarity of the substrate-binding site of Ord1 with other previously assigned prenyltransferases showed that the Ord1 residues that were characteristically located close to the pyrophosphate in FSPP (i.e., Y215 and Q216) and the pseudo-SARM (i.e., D219, D220, and A223) are shared with the prenyltransferase XiaM (Figure S1), suggesting that Ord1 may accommodate an indole as a prenyl acceptor substrate. We therefore soaked Ord1 crystals in buffer containing FSPP and indole to obtain the structure of the ternary complex. Unfortunately, only the binary complex structure of Ord1-FSPP was obtained. Therefore, molecular simulations of the docking of indole to apo-Ord1 were carried out. These simulations predicted an interaction between the N1 atom of indole and the Oε atom of Ord1 Q216 via a hydrogen bond (Figure 3D).

### In Vitro Analysis of the Enzymatic Activity of Ord1

The product of Ord1 was investigated by using HPLC analysis of enzyme reaction mixtures. The product of Ord1 eluted at 38 min, but no product was formed by heat-treated Ord1 (Figure 4A). The enzyme reaction product was identified using LC-MS and exhibited an *m*/*z* value of 322.25299, with an MS tolerance of 1.9 ppm against the theoretical *m*/*z* value of 3-farnesylindole (Figure S3). HPLC analysis also showed that farnesylindole was produced more efficiently than geranylindole, suggesting that FPP is the preferred prenyl donor substrate (Figure S4). This observation is consistent with the oridamycin scaffold isolated from the same *Streptomyces* strain, which is likely derived from a farnesyl moiety.^33^ To further investigate the substrate specificity of Ord1, 1-methylindole was used as a prenyl acceptor substrate. However, no product was detected by using HPLC (Figure 4B), suggesting that the hydrogen atom at position N1 in the indole is crucial for catalysis by Ord1.

**Figure 4:**
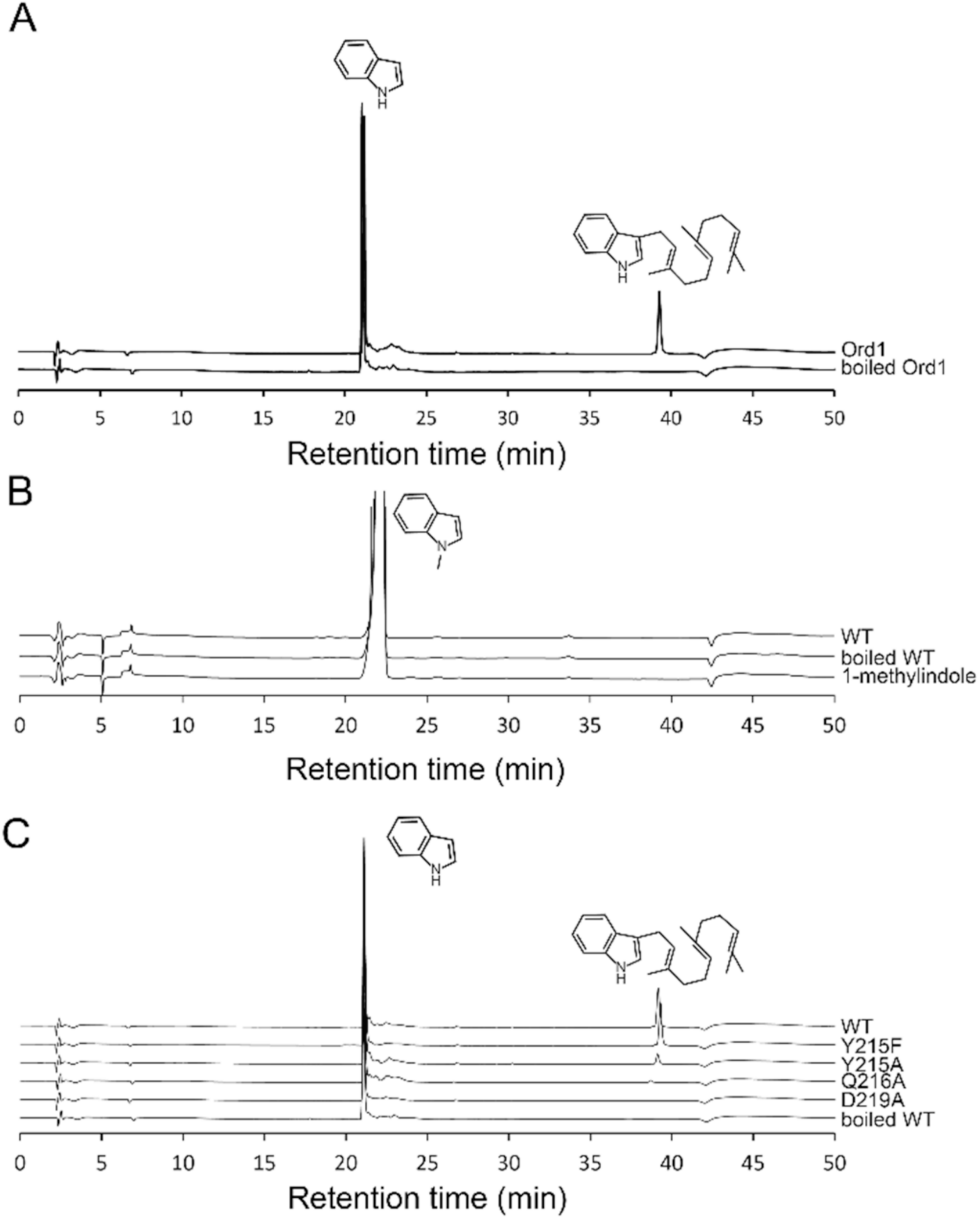
Enzyme reaction products of the Ord1 and its mutant enzymes. Enzyme reaction products of (A) indole and (B) 1-methylindole as the acceptor substrate by Ord1 and boiled Ord1 are shown. (C) Enzyme reaction products of indole catalyzed by Ord1 WT, Y215F, Y215A, Q216A, and D219A mutant enzymes are shown.

### Structure-Based Mutagenesis of Ord1

Based on the above observations and simulations, we constructed an Ord1 site-directed mutant, Q216A. The Q216A mutant generated product in the presence of FPP and indole; however, the yield was significantly decreased (4.2%) compared with that of the WT enzyme, resulting in a nearly complete loss of activity (Figure 4C, Table S3). Gln was not deprotonated spontaneously under the reaction conditions of pH 8.0; consequently, Gln was not available to directly abstract the proton from the N1 atom of indole (Figure 3D). Initiation of the catalytic reaction requires a certain active residue to nucleophilically attack Q216. A detailed analysis of the amino acids around Q216 showed that the Oδ atom of D219 is located one helical pitch downstream of Q216 and within a distance suitable for the formation of a hydrogen bond with the Nε atom of Q216 (Figure 3D). We therefore constructed an Ord1 D219A mutant to clarify whether D219 is involved in the catalytic reaction. Incubation of the D219A mutant, FPP, and indole resulted in no product formation (Figure 4C). As flipping of the side chain of Y215 was observed in the FSPP complex, this flipping was considered necessary for the enzyme to recognize and accommodate an indole near the reaction site. Therefore, Ord1 Y215A and Y215F mutants were also constructed, and their activity was evaluated. The relative activity of the Ord1 Y215F mutant was 105% of the activity of WT Ord1 (Figure 4C and Table S3). In contrast to the activity of the Y215F mutant, the activity of Ord1 Y215A relative to WT Ord1 was reduced by >80% (Figure 4C and Table S3), suggesting that the aromatic ring at position 215 is in an appropriate position to form a π–π stacking interaction between Tyr/Phe and the indole, stabilizing the prenyl acceptor substrate in the proper position.

### Structure of Ord1 Q216A with the Substrate and Ord1 D219A

To confirm that the changes in the activity of the mutant enzymes described above were not due to the collapse of the protein structure derived from the substitution of amino acids, the crystal structures of Ord1 Q216A bound with FSPP and the D219A mutant were determined at a resolution of 2.22 and 2.80 Å, respectively (Figure 5A,B). The Q216A mutant accommodated FSPP in the cavity, as seen with the WT enzyme, and superimposed well with WT Ord1 bound to FSPP, with an RMSD of 0.52 Å (Figure 5C). No difference was found, except at the position where the bulkier residue Gln was substituted with the smaller residue Ala (Figure 5C). The manner in which Mg^2+^ ions were coordinated was very similar to that of WT Ord1; that is, only one Mg^2+^ ion was coordinated with D85, D89, and the pyrophosphate of FPP (Figure 5C). These results indicate that the Q216A mutant retains the interaction with FSPP independent of the structural change yet fails to produce a product with the indole. The Oε atom of Q216 abstracts the hydrogen from the N1 atom of indole, thereby catalyzing the prenylation reaction. Furthermore, the RMSD value for the superimposition of the Ord1 D219A mutant and WT Ord1 was 0.41 Å (Figure 5B), indicating no significant difference (Figure 5B,D). Thus, we propose that the enzymatic mechanism of all-α-helical prenyltransferases proceeds as follows: the Oδ atom of D219 triggers a nucleophilic attack to initiate the catalytic reaction, leading to the sequential transfer of the prenyl chain to indole (Figure 5E).

**Figure 5:**
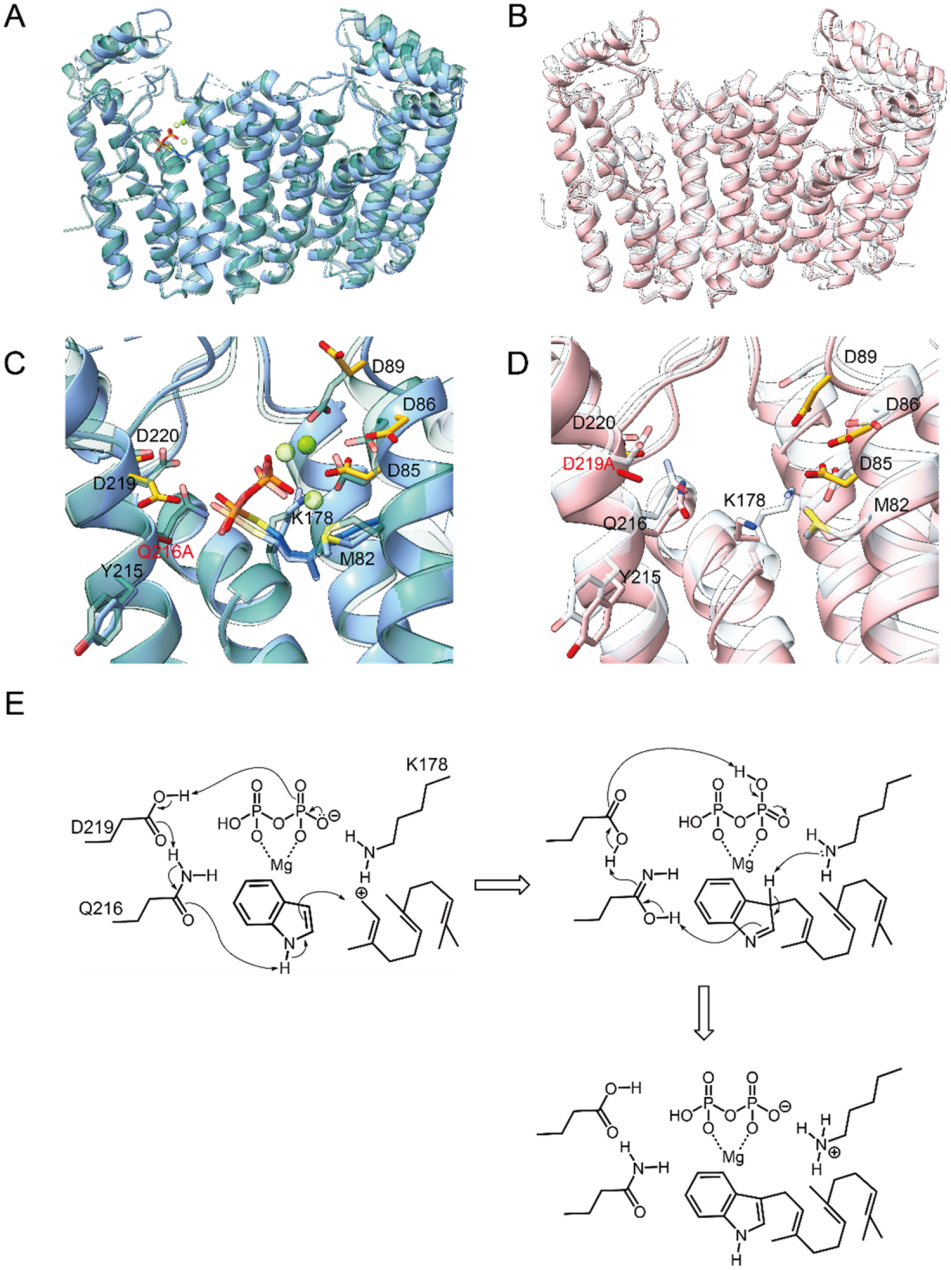
Crystal structures of the Ord1 Q216A and D219A mutants. The overall structures of (A) Ord1 Q216A-FSPP (blue) superimposed onto Ord1-FSPP (green) and (B) Ord1 D219A (pink) superimposed onto Ord1 (white) are illustrated as ribbon diagrams. Comparisons of the structures of (C) Ord1-FSPP and Ord1 Q216A-FSPP and (D) Ord1 apo and Ord1 D219A are shown. (E) Predicted catalytic mechanism of Ord1.

## Conclusions

Terpenoids represent the largest and most widely distributed family of natural products.^3^ This enormous diversity derives from the multitude of scaffolds generated by enzymes of the prenylsynthase family. In this study, we determined for the first time the structure of the prenyltransferase, which belongs to the all-α-helical prenylsynthase superfamily. Analyses of the X-ray crystal structures and activities of Ord1 and various mutants revealed that Q216 plays a significant role by interacting with the N1 atom of indole, whereas Y215 is involved in recognizing and placing the substrate in the proper spatial position for the catalytic reaction. Our results also suggest that the Oδ atom of D219 initiates the catalytic reaction by promoting a nucleophilic attack mediated by the side chain of Q216. This process involves abstraction of the hydrogen from the N1 atom of indole by the Oε atom of Q216, followed by the transfer of the prenyl chain to the indole ring. Ord1 utilizes the FARM to bind the pyrophosphate group, as observed in known prenylsynthases (Figures 1D and 3C). In contrast to the FARM residues, D219 in the pseudo-SARM of Ord1 directly interacts with the pyrophosphate group, compensating for the absence of the SARM-coordinated metal ion that is typically observed in prenylsynthases (Figures 1D and 3C). It results in a distinct mode of FPP binding between prenylsynthases and prenyltransferases. Notably, prenylsynthases do not catalyze a direct nucleophilic attack on the substrate but instead provide an environment conducive to the formation of the allylic carbocation.^47–49^ In contrast, no condensation of isoprene units was observed in a mixture of Ord1, a prenylpyrophosphate (GPP or FPP), and IPP (Figure S4A), indicating that Ord1 does not promote allylic carbocation formation. This represents a key mechanistic distinction and supports the conclusion that Ord1 catalyzes a direct nucleophilic attack. Furthermore, a recent in silico model of the prenyltransferase PalQ^26^ proposed a reaction mechanism involving a nucleophilic interaction between D201 (corresponding to D219 in Ord1) and Q197 (corresponding to Q216 in Ord1), along with interactions between the conserved Oε atom of Q197 and the N1 atom of the indole ring. Our experimentally derived conclusions are consistent with this proposed mechanism by the in silico model. Additionally, no cyclized product derived from the farnesyl group was observed in reactions containing Ord1 and FPP, suggesting that the activity of Ord1 lacks cyclase activity. These findings enhance our understanding of the catalytic mechanism of all-α-helical prenyltransferases and may contribute to the rational design of artificial enzymes based on prenyltransferases for the enzymatic biosynthesis of structurally diverse and useful compounds.

## Supplementary Material



## Data Availability

The raw data generated in this study have been deposited in the Zenodo 10.5281/zenodo.15582090 and available from the corresponding authors upon request.

## Notes

Multiple sequence alignment of prenyltransferases and prenylsynthases; close-up view of Y215 side chain flip in the Ord1-FSPP crystal; LC-MS analysis of the products of Ord1 enzyme assays; HPLC and MS analyses of reaction products; primers used to generate Ord1 mutations; summary of crystallization conditions, data collection, and refinement statistics; and relative activities of Ord1 and mutant enzymes (PDF)

## Abbreviations

ARM: aspartate-rich motif
DMAPP: dimethylallyl pyrophosphate
FARM: first aspartate-rich motif
FPP: farnesyl pyrophosphate
FSPP: farnesyl thiolodiphosphate
GGPP: geranylgeranyl pyrophosphate
GPP: geranyl pyrophosphate
IPP: isopentenyl pyrophosphate
IPTG: isopropyl-β-d-1-thiogalactopyranoside
SARM: second aspartate-rich motif
