# Supplementary material for: Structure–Activity Relationship of an All-α-helical Prenyltransferase Reveals the Mechanism of Indole Prenylation
Source: Biochemistry. 2025 Sep 19;64(19):4196–205. doi: 10.1021/acs.biochem.5c00329 (PMC12510300; doi:10.1021/acs.biochem.5c00329)
Supplement: Supplementary file 1 [file bi5c00329_si_001.pdf]

Supporting Information

Structure-activity relationship of an all-α-helical prenyltransferase reveals the mechanism of indole prenylation

Takumi Oshiro[1], Shuta Uehara[1], Arisa Suto[1], Yoshikazu Tanaka[2], Takuya Ito[3], Yoshio Kodera[1,4], Takashi Matsui[1,4,*]

[1]Department of Physics, School of Science, Kitasato University, Sagamihara, Kanagawa 252-0373, Japan

[2]Graduate School of Life Science, Tohoku University, Sendai, Miyagi 980-8577, Japan

[3]Faculty of Pharmacy, Osaka Ohtani University, Tondabayashi, Osaka 585-8540, Japan

[4]Center for Disease Proteomics, School of Science, Kitasato University, Sagamihara, Kanagawa 252-0373, Japan

*Correspondence: Dr. Takashi Matsui, e-mail: matsui@kitasato-u.ac.jp, Postal address: 1-15-1 Kitasato, Minami-ku, Sagamihara, Kanagawa 252-0373, Japan

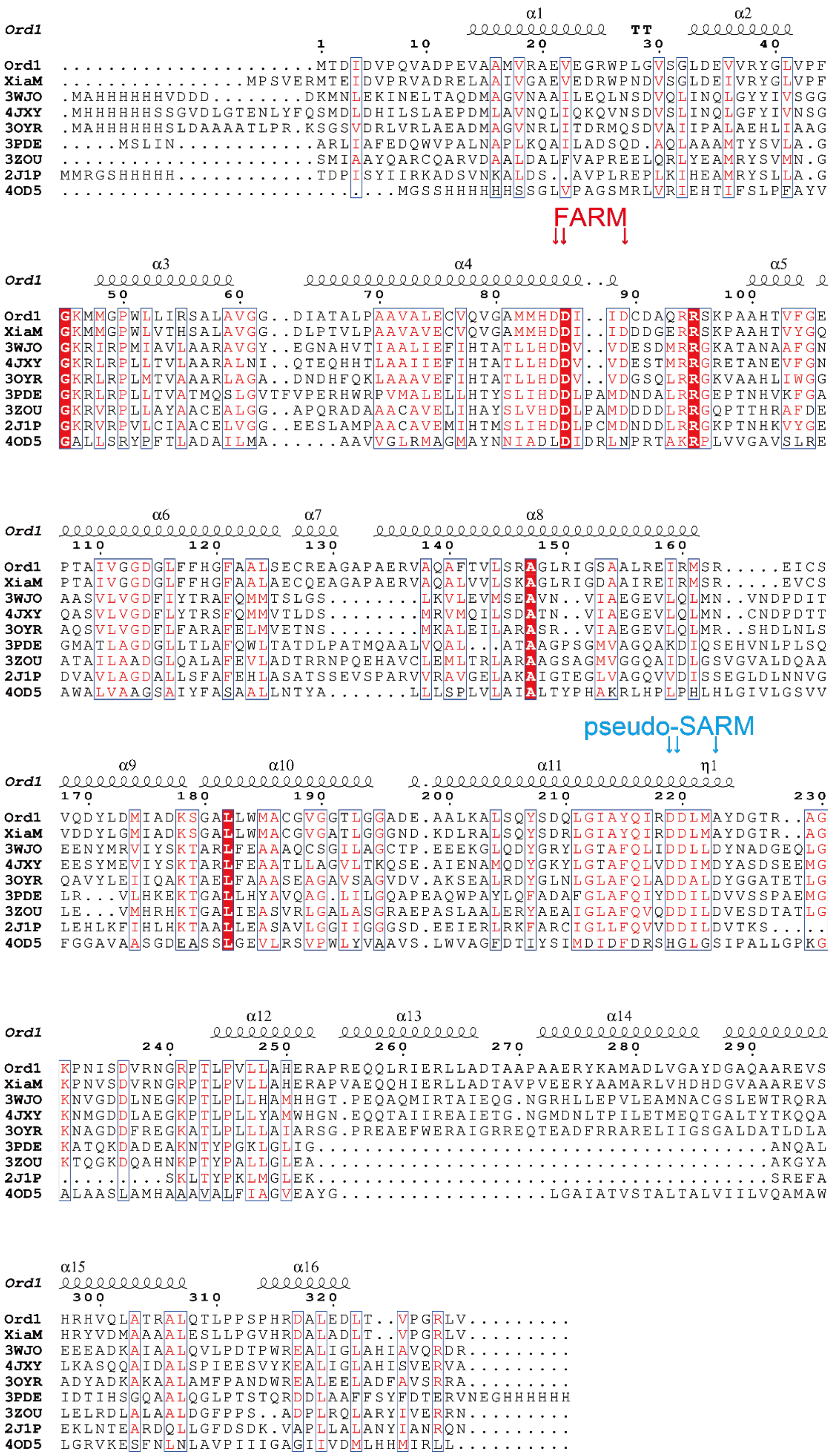
α1 α2 TT
1 10 20 30 40
Ord1 ......................MTDIDVPQVADPEVAAMVRAEVEGRWPLGVSGLDEVVRYGLVPF
XiaM ................MPSVERMTEIDVPRVADRELAAIVGAEVEDRWPNDVSGLDEIVRYGLVPF
3WJO .MAHHHHHHVDDD........DKMNLEKINELTAQDMAGVNAAILEQLNSDVQLINQLGYYIVSGG
4JXY .MHHHHHHSSGVDLGTENLYFQSMDLDHILSLAEPDMLAVNQLIQKQVNSDVSLINQLGFYIVNSG
3OYR .MAHHHHHHSLDAAAATLPR.KSGSVDRLVRLAEADMAGVNRLITDRMQSDVAIIPALAEHLIAAG
3PDE .....MSLIN............ARLIAFEDQWVPALNAPLKQAILADSQD.AQLAAAMTYSVLA.G
3ZOU ......................SMIAAYQARCQARVDAALDALFVAPREELQRLYEAMRYSVMN.G
2J1P MMRGSHHHHH............TDPISYIIRKADSVNKALDS..AVPLREPLKIHEAMRYSLLA.G
4OD5 ..........................MGSSHHHHHHSSGLVPAGSMRLVRIEHTIFSLPFAYV
FARM
α3 α4 α5
50 60 70 80 90 100
Ord1 GKMMGPWLLIRSALAVGG..DIATALPAAVALECVQVGAMMHDDI..IDCDAQRRSKPAAHTVFGE
XiaM GKMMGPWLVTHSALAVGG..DLPTVLPAAVAVECVQVGAMMHDDI..IDDDGERRSKPAAHTVYGQ
3WJO GKRIRPMIAVLAARAVGY..EGNAHVTIAALIEFIHTATLLHDDV..VDESDMRRGKATANAAFGN
4JXY GKRLRPLLTVLAARALNI..QTEQHHTLAAIIEFIHTATLLHDDV..VDESTMRRGRETANEVFGN
3OYR GKRLRPLMTVAAARLAGA..DNDHFQKLAAAVEFIHTATLLHDDV..VDGSQLRRGKVAAHLIWGG
3PDE GKRLRPLLTVATMQSLGVTFVPERHWRPVMALELLHTYSLIHDDLPAMDNDALRRGEPTNHVKFGA
3ZOU GKRVRPLLAYAACEALGG..APQRADAACAVELIHAYSLVHDDLPAMDDDDLRRGQPTTHRAFDE
2J1P GKRVRPVLCIAACELVGG..EESLAMPAACAVEMIHTMSLIHDDLPCMDNDDLRRGKPTNHKVYGE
4OD5 GALLSRYPFTLADAILMA......AAVVGLRMAGMAYNNIADLDIDRLNPRTAKRPLVVGAVSLRE
α6 α7 α8
110 120 130 140 150 160
Ord1 PTAIVGGDGLFFHGFAALSECREAGAPAERVAQAFTVLSRAGLRIGSAALREIRMSR.....EICS
XiaM PTAIVGGDGLFFHGFAALAECQEAGAPAERVAQALVVLSKAGLRIGDAAIREIRMSR.....EVCS
3WJO AASVLVGDFIYTRAFQMMTSLGS........LKVLEVMSEAVN..VIAEGEVLQLMN..VNDPDIT
4JXY QASVLVGDFLYTRSFQMMVTLDS........MRVMQILSDATN..VIAEGEVLQLMN..CNDPDTT
3OYR AQSVLVGDFLFARAFELMVETNS........MKALEILARASR..VIAEGEVLQLMR..SHDLNLS
3PDE GMATLAGDGLLTLAFQWLTATDLPATMQAALVQAL...ATAAGPSGMVAGQAKDIQSEHVNLPLSQ
3ZOU ATAILAADGLQALAFEVLADTRRNPQEHAVCLEMLTRLARAAGSAGMVGGQAIDLGSVGVALDQAA
2J1P DVAVLAGDALLSFAFEHLASATSSEVSPARVVRAVGELAKAIGTEGLVAGQVVDISSEGLDLNNVG
4OD5 AWALVAAGSAIYFASAALLNTYA.......LLLSPLVLAIALTYPHAKRLHPLPHLHLGIVLGSVV
pseudo-SARM
α9 α10 α11 η1
170 180 190 200 210 220 230
Ord1 VQDYLDMIADKSGALLWMACGVGGTLGGADE.AALKALSQYSDQLGIAYQIRDDLMAYDGTR..AG
XiaM VDDYLGMIADKSGALLWMACGVGATLGGGND.KDLRALSQYSDRLGIAYQIRDDLMAYDGTR..AG
3WJO EENYMRVIYSKTARLFEAAAQCSGILAGCTP.EEEKGLQDYGRYLGTAFQLIDDLLDYNADGEQLG
4JXY EESYMEVIYSKTARLFEAATLLAGVLTKQSE.AIENAMQDYGKYLGTAFQLVDDIMDYASDSEEMG
3OYR QAVYLEIIQAKTAELFAAASEAGAVSAGVDV.AKSEALRDYGLNLGLAFQLADDALDYGGATETLG
3PDE LR...VLHKEKTGALLHYAVQAG.LILGQAPEAQWPAYLQFADAFGLAFQIYDDILDVVSSPAEMG
3ZOU LE...VMHRHKTGALIEASVRLGALASGRAEPASLAALERYAEAIGLAFQVQDDILDVESDTATLG
2J1P LEHLKFIHLHKTAALLEASAVLGGIIGGGSD.EEIERLRKFARCIGLLFQVVDDILDVTKS.....
4OD5 FGGAVAASGDEASSLGEVLRSVPWLYVAAVS.LWVAGFDTIYSIMDIDFDRSHGLGSIPALLGPKG
α12 α13 α14
240 250 260 270 280 290
Ord1 KPNISDVRNGRPTLPVLLAHERAPREQQLRIERLLADTAAPAAERYKAMADLVGAYDGAQAAREVS
XiaM KPNVSDVRNGRPTLPVLLAHERAPVAEQQHIERLLADTAVPVEERYAAMARLVHDHDGVAAAREVS
3WJO KNVGDDLNEGKPTLPLLHAMHHGT.PEQAQMIRTAIEQG.NGRHLLEPVLEAMNACGSLEWTRQRA
4JXY KNMGDDLAEGKPTLPLLYAMWHGN.EQQTAIIREAIETG.NGMDNLTPILETMEQTGALTYTKQQA
3OYR KNAGDDFREGKATLPLLLAIARSG.PREAEFWERAIGRREQTEADFRRARELIIGSGALDATLDLA
3PDE KATQKDADEAKNTYPGKLGLIG..............................ANQAL
3ZOU KTQGKDQAHNKPTYPALLGLEA..............................AKGYA
2J1P ........SKLTYPKLMGLEK...............................SREFA
4OD5 ALAASLAMHAAAVALFIAGVEAYG..................LGAIATVSTALTALVIILVQAMAW
α15 α16
300 310 320
Ord1 HRHVQLATRALQTLPPSPHRDALEDLT..VPGRLV.........
XiaM HRYVDMAAAALESLLPGVHRDALADLT..VPGRLV.........
3WJO EEEADKAIAALQVLPDTPWREALIGLAHIAVQRDR.........
4JXY LKASQQAIDALSPIEESVYKEALIGLAHISVERVA.........
3OYR ADYADKAKAALAMFPANDWREALEELADFAVSRRA.........
3PDE IDTIHSGQAALQGLPTSTQRDDLAAFFSYFDTERVNEGHHHHHH
3ZOU LELRDLALAALDGFPPS..ADPLRQLARYIVERRN.........
2J1P EKLNTEARDQLLGFDSDK.VAPLLALANYIANRQN.........
4OD5 LGRVKESFNLNLAVPIIIGAGIIVDMLHHMIRLL..........

Supplementary Figure S1. Multiple sequence alignment of prenyltransferases and prenylsynthases.

Sequence alignment of two all-α-prenyltransferases (Ord1 and XiaM), six prenylsynthases (3WJO, 4JXY, 3OYR, 3PDE, 3ZOU, and 2J1P), and Ubi-A prenyltransferase (4OD5) using Clustal OMEGA (1.2.4),[44] visualized using ESPript 3.0 (https://endscript.ibcp.fr). [45] Conserved sequences are colored red. Secondary structure elements as identified in the crystal structure of Ord1 molA. FARM and pseudo-SARM sequences are indicated by red and blue arrows, respectively. XiaM, 3WJO, 4JXY, 3OYR, 3PDE, 3ZOU, and 2J1P shared 79.6, 26.6, 27.6, 30.4, 29.2, 31.9, and 31.3% identity with Ord1, respectively. 4OD5 did not exhibit significant similarity with Ord1.

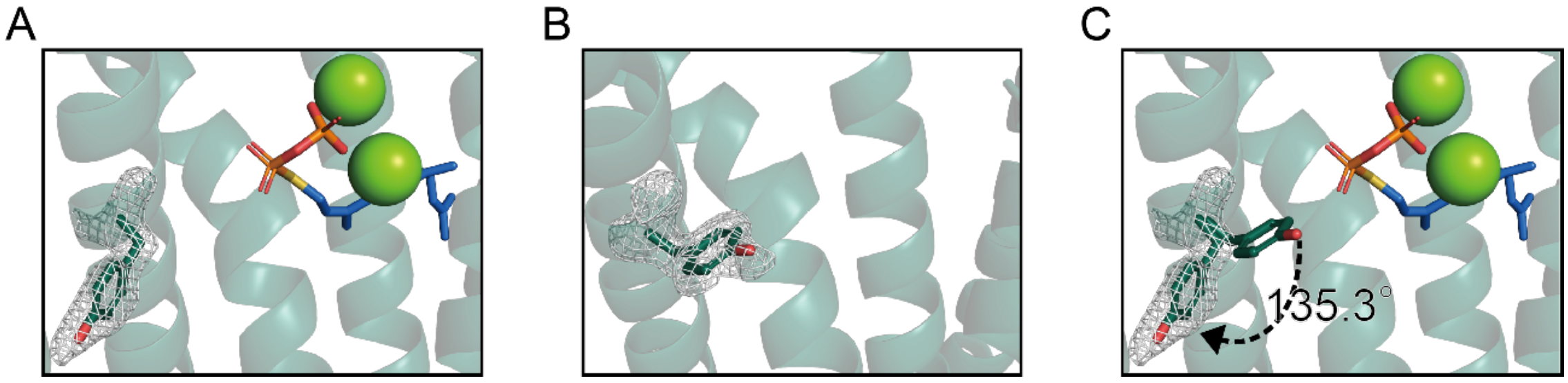

Supplementary Figure S2. Close-up view of Y215 side chain flip in theOrd1–FSPP crystal.

Substrate-binding sites in (A) the FSPP-bound form (molecule B) and (B) the apo form (molecule A). (C) Flipping of the Y215 side chain shown by superimposing molecules A and B. The $2F_o$–F_c electron density map contoured at 1.0 σ for the Y215 side chain is shown as a white mesh.

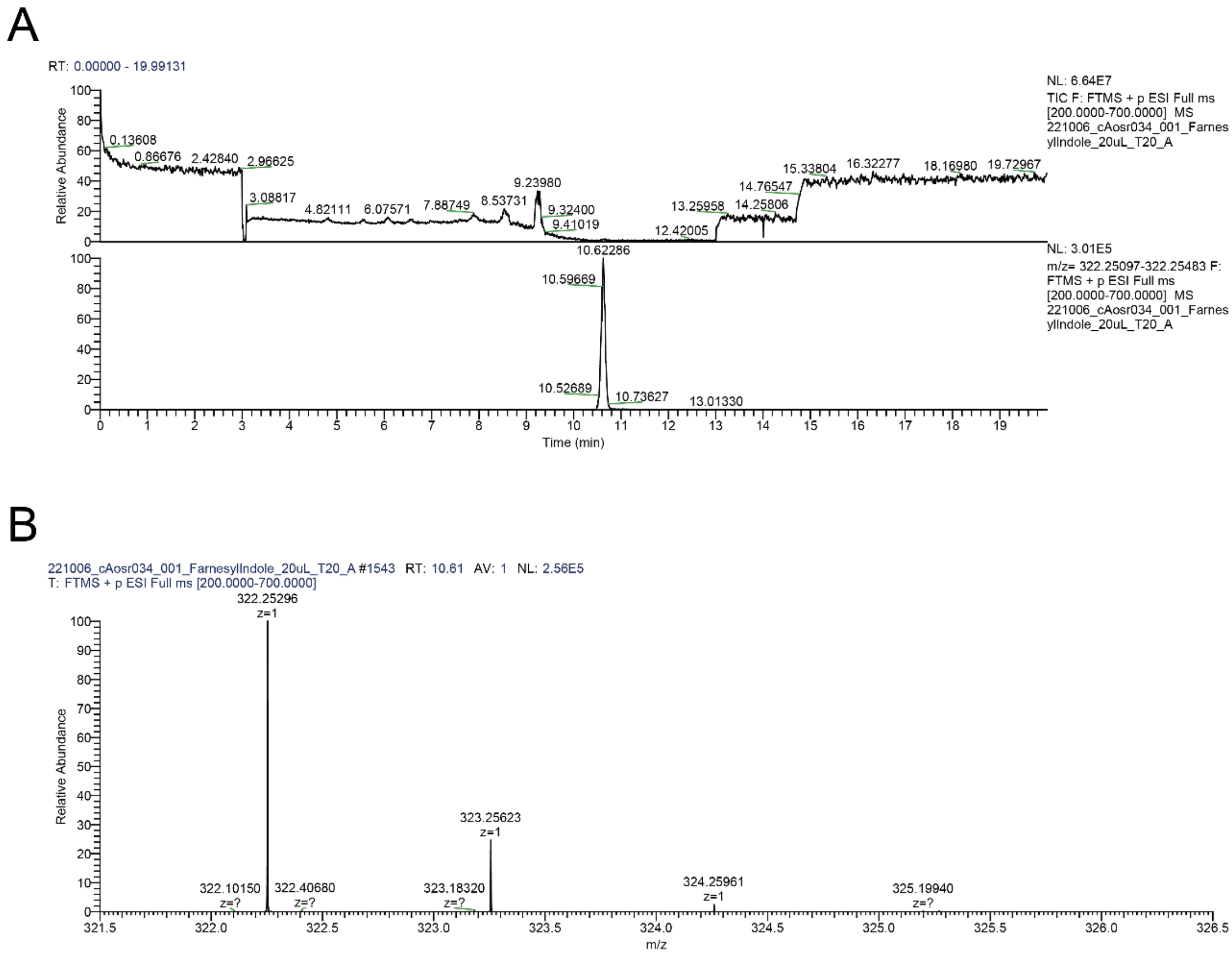

Supplementary Figure S3. LC-MS analysis of the products of Ord1 enzyme assays. (A) Total ion chromatogram ranging from 200-700 *m/z* is shown (upper trace). Extracted ion chromatogram at *m/z* 322.25299 with a mass tolerance 6 ppm is also shown (lower trace). (B) Mass spectrum of the peak eluting at a retention time of 10.61 min is shown.

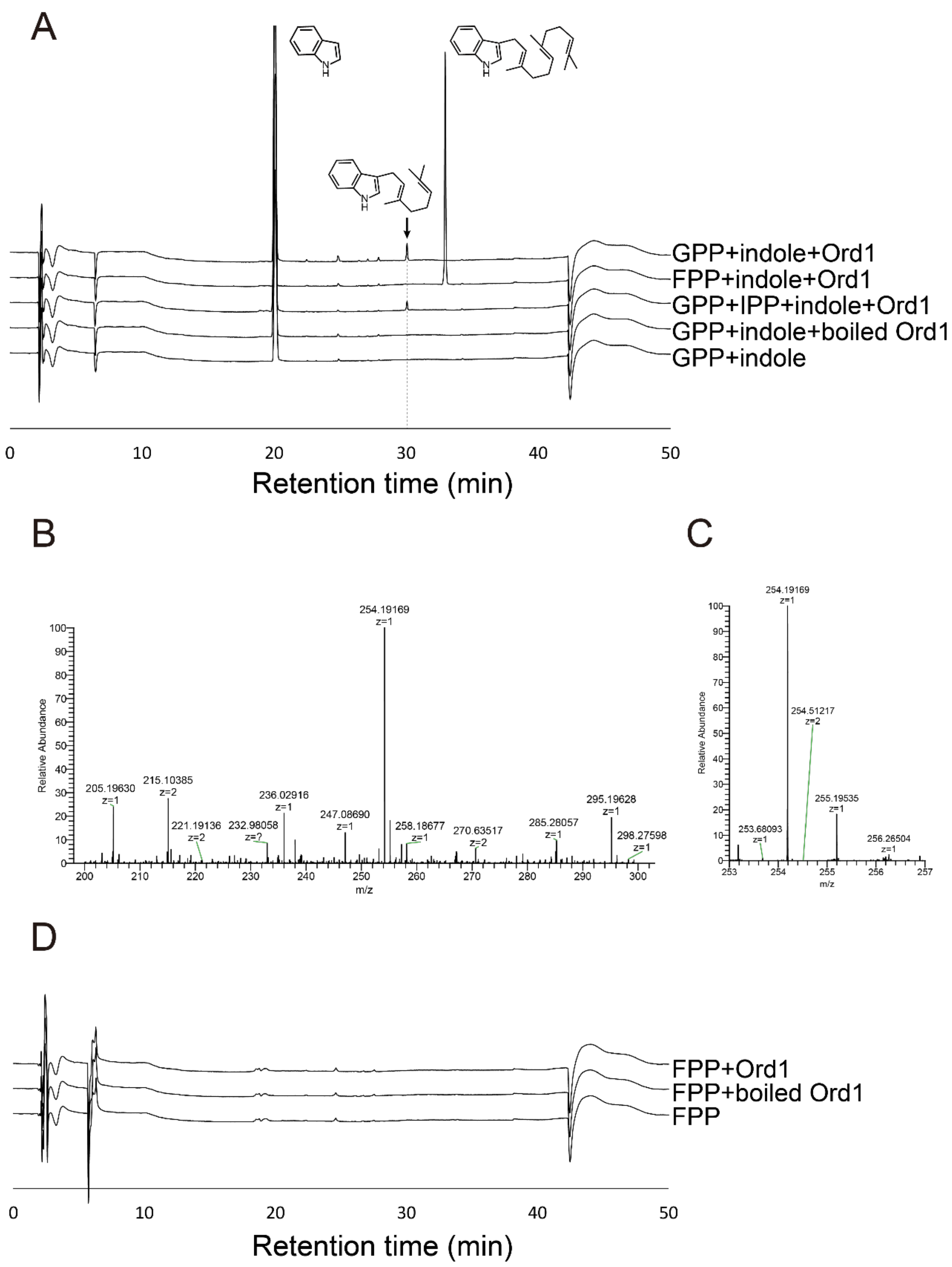

Supplementary Figure S4. HPLC and MS analyses of reaction products.

(A) Although no elongation activity was observed in the enzymatic reaction with Ord1, a

small amount of geranylindole was detected in the mixture containing GPP. This result suggests that GPP can serve as a substrate for Ord1; however, farnesylindole was produced more efficiently than geranylindole. No enzymatic products were observed when boiled Ord1 was used, indicating that prenylindole production depends on the presence of catalytically active Ord1. (B) Mass spectrum corresponding to the reaction product formed in the reaction with GPP and indole shown in panel A. The MS spectrum of the product generated by enzymatic reaction of Ord1 with GPP, IPP, and indole (corresponding to the peak shown in panel A) exhibits an *m*/*z* value of 254.19169 which is consistent with the theoretical *m*/*z* value of 3-geranylindole ($[M+H]^+$; calculated for $C_{18}H_{24}N^+$, 254.19032). (C) Close-up view of the mass spectrum ranging from 253 to 257. The monoisotopic peak (theoretical m/z of 254.19032, 100% relative intensity) and its isotope peak (theoretical m/z of 255.19368, 19.5% relative intensity) of 3-geranylindole were clearly observed. (D) No cyclization activity was observed. No products were detected under cyclization conditions using FPP as the substrate.

Supplementary Table S1. Primers used to generate Ord1 mutations.

Primer name	Sequence (5' to 3')
Y215A-F	AGCTGGGCATCGCCGCCCAGATCCGTGACG
Y215A-R	CGTCACGGATCTGGGCGGCGATGCCCAGCT
Y215F-F	AGCTGGGCATCGCCTTCCAGATCCGTGACG
Y215F-R	CGTCACGGATCTGGAAGGCGATGCCCAGCT
Q216A-F	TGGGCATCGCCTACGCGATCCGTGACGACC
Q216A-R	GGTCGTCACGGATCGCGTAGGCGATGCCCA
D219A-F	ACGCCATGAGGTCGGCACGGATCTGGTAG
D219A-R	CTACCAGATCCGTGCCGACCTCATGGCGT

The sequence belonging to the mutate sequence is underlined.

Supplementary Table S2. Summary of crystallization conditions, data collection, and refinement statistics[a].

	Ord1 SeMet	Ord1	Ord1-FSPP	Ord1 Q216A-FSPP	Ord1 D219A
**Sample conditions**					
Reservoir condition	0.2 M Li_2SO_4, 0.1 M Bis-Tris (pH 5.5), 25% PEG3350	0.2 M $(NH_4)_2SO_4$, 0.1 M Bis-Tris (pH 5.5), 25% PEG3350	0.2 M $(NH_4)_2SO_4$, 0.1 M Bis-Tris (pH 5.5), 25% PEG3350	0.24 M Li_2SO_4, 0.1 M Bis-Tris (pH 5.5), 15% PEG3350	0.2 M $(NH_4)_2SO_4$ 0.1 M Bis-Tris (pH 5.5), 25% PEG3350
Soaking	-	-	1 mM FSPP 5 mM indole 5 mM $MgSO_4$	1 mM FSPP 5 mM indole 5 mM $MgSO_4$	1 mM FSPP 5 mM indole 5 mM $MgSO_4$
Cryoprotectant	10% Ethylene glycol	10% Ethylene glycol	10% Ethylene glycol	10% Ethylene glycol	10% Ethylene glycol
**Data collection**					
Beamline	PF BL-5A	PF BL-1A	PF BL-1A	PF BL-1A	PF BL-17A
Wavelength (Å)	0.97922	1.04500	1.04500	1.04500	0.98000
Space group	*P*1	*P*1	*P*1	*P*1	*P*1
a, b, c (Å)	59.4, 72.9, 84.4	59.0, 72.2, 84.2	59.1, 72.6, 84.2	58.9, 71.9, 84.0	58.9, 72.4, 83.8
α, β, γ (°)	94.3, 69.5, 90.0	86.0, 69.5, 90.0	94.0, 69.5, 90.0	86.3, 69.4, 90.0	93.5, 110.5, 90.1
Resolution range (Å)	50.0-1.78 (1.89-1.78)	50.0-2.10 (2.22-2.10)	50.0-2.00 (2.12-2.00)	50.00-2.220 (2.33-2.22)	50.0-2.80 (2.97-2.80)
Completeness (%)	95.8 (93.7)	97.6 (96.0)	97.6 (96.6)	97.9 (97.0)	97.8 (97.2)
$\langle I/\sigma(I) \rangle$	31.53 (2.85)	10.58 (2.25)	12.71 (1.75)	11.37 (2.02)	11.83 (3.09)
R_{merge}[b] (%)	3.0 (66.8)	6.3 (52.3)	5.2 (70.6)	6.7 (57.3)	11.8 (71.2)
$CC_{1/2}$[c]	1.000 (0.880)	0.998 (0.774)	0.999 (0.746)	0.999 (0.746)	0.996 (0.771)
Multiplicity	7.2 (7.3)	3.2 (3.4)	3.6 (3.7)	3.6 (3.7)	3.6 (3.7)
Total reflections	1,739,580 (281,067)	240,896 (39,866)	312,316 (51,426)	232,848 (38,558)	111,425 (18,250)
No. of unique reflections	242,171 (38,269)	74,272 (11,833)	86,258 (13,824)	91,888 (14,660)	31,241 (4,990)
**Refinement collection**					
Resolution range (Å)		44.40-2.10	55.62-2.00	44.26-2.22	44.35-2.80
R_{work}[d] (%)		22.87	23.82	23.57	22.40
R_{free}[e] (%)		24.59	27.22	25.34	25.39
RMS (bonds) (Å)		0.004	0.008	0.003	0.004
RMS (angles) (°)		0.7	1.0	0.6	0.7
Twin fraction		0.500 (*h*, -*k*, *h*-*l*)	0.500 (*h*, -*k*, *h*-*l*)	0.500 (*h*, -*k*, *h*-*l*)	0.490 (*h*, -*k*, -*h*-*l*)
Clash score		8.43	9.52	9.70	13.41
**Number of atoms**					

Protein	8506	8078	8348	8567
Ligand	35	57	49	1
Water	173	210	53	8
***B*-factor (Å²)**				
Protein	51	48	53	68
Ligand	51	44	60	43
Water	40	43	40	51
**Ramachandran plot**				
Ramachandran favored (%)	98.65	98.95	98.72	98.66
Ramachandran allowed (%)	1.36	0.95	1.28	1.34
Ramachandran outliers (%)	0.09	0.10	0	0

[a] Values in parentheses are for the highest resolution shell.

[b] $R_{merge} = \Sigma_h\Sigma_j |<I>_h - I_{h,j}|/\Sigma_h\Sigma_j I_{h,j}$, where $<I>_h$ is the mean intensity of symmetry-equivalent reflections.

[c] $CC_{1/2}$ is the Pearson correlation coefficient for a random half of the data, the two numbers represent the lowest and highest resolution shell, respectively.

[d] $R_{work} = \Sigma|F_{obs} - F_{cal}|/\Sigma F_{obs}$, where F_{obs} and F_{cal} are observed and calculated structure factor amplitudes, respectively.

[e] R_{free} value was calculated for R factor, using only an unrefined subset of reflection data.

Supplementary Table S3. Relative activities of Ord1 and mutant enzymes.

Mutants	Relative activity (%)
WT	100.0 ± 5.1
Y215F	104.9 ± 7.5
Y215A	11.2 ± 1.4
Q216A	4.2 ± 0.2
D219A	N/D

Values are means ± standard deviation (n = 3). N/D means not detected.
